# A Synthetic Biology Perspective on the Bioengineering Tools for an Industrial Microalga: *Euglena gracilis*

**DOI:** 10.3389/fbioe.2022.882391

**Published:** 2022-04-06

**Authors:** Zhenfan Chen, Jiayi Zhu, Ming Du, Zixi Chen, Qiong Liu, Hui Zhu, Anping Lei, Jiangxin Wang

**Affiliations:** ^1^ College of Food Engineering and Biotechnology, Hanshan Normal University, Chaozhou, China; ^2^ Shenzhen Key Laboratory of Marine Bioresource and Eco-Environmental Science, Shenzhen Engineering Laboratory for Marine Algal Biotechnology, Guangdong Provincial Key Laboratory for Plant Epigenetics, College of Life Sciences and Oceanography, Shenzhen University, Shenzhen, China; ^3^ Key Laboratory of Optoelectronic Devices and Systems of Ministry of Education and Guangdong Province, College of Optoelectronic Engineering, Shenzhen University, Shenzhen, China; ^4^ Shenzhen-Hong Kong Institute of Brain Science, Shenzhen, China

**Keywords:** *Euglena gracilis*, genetic transformation, biotechnology, CRISPR, RNAi

## Abstract

*Euglena* is a genus of single-celled eukaryotes that show both plant- and animal-like characteristics. *Euglena gracilis*, a model species, is of great academic interest for studying endosymbiosis and chloroplast development. As an industrial species, *E. gracilis* is also of primary biotechnological and economic importance as high value-added food, medicine, and cosmetic and high-quality feedstock for jet-fuel production because of its cells containing many high-value products, such as vitamins, amino acids, pigments, unsaturated fatty acids, and carbohydrate paramylon, as metabolites. For more than half a century, *E. gracilis* has been used as an industrial biotechnology platform for fundamental biology research, mainly exploring relevant physiological and biochemical method studies. Although many researchers focused on genetic engineering tools for *E. gracilis* in recent years, little progress has been achieved because of the lack of high-quality genome information and efficient techniques for genetic operation. This article reviewed the progress of the genetic transformation of *E. gracilis*, including methods for the delivery of exogenous materials and other advanced biotechnological tools for *E. gracilis*, such as CRISPR and RNA interference. We hope to provide a reference to improve the research in functional genomics and synthetic biology of *Euglena*.

## Introduction

*Euglena gracilis* (*E. gracilis*) is a single-celled eukaryotic alga without a cell wall but has flagella on the top of the cell. It majorly lives in freshwater, is distributed worldwide, and could bloom in ponds, rivers, wastewater, etc. *E. gracilis* exhibits both animal and plant features with various modes of nutrition, including autotrophy, heterotrophy, and mixotrophy (Torihara and Kishimoto, 2015). As a model microorganism, *E. gracilis* has been studied extensively in many aspects, such as the development of secondary endosymbiotic chloroplasts, bioremediation of environmental pollutions, and decreasing carbon dioxide emissions, especially the improved harvest of high-value products that people in the market favor (Klinthong et al., 2015; Gissibl et al., 2019; Tahira et al., 2019). *E. gracilis* cells contain linear polysaccharide paramylon, wax esters, vitamins, and amino acids and have immense commercial importance (Gissibl et al., 2019). For example, paramylon, with a specific molecular structure (*β*-1, 3-glucan), can be used as a functional food ingredient. The content of paramylon in *E. gracilis* is much higher than that in other species such as fungi. Yasuda et al. (2018) reported that the maximum dry weight of the cell in *E. gracilis* can reach up to 70%. *E. gracilis* can not only be valid for the production of nutraceuticals and cosmeceuticals but also be used in biofuels. The glucan crystals produced from *E. gracilis* can be transformed into wax esters under anerobic conditions. They have relatively lower freezing points and are appropriate for being a feedstock of carbon chemical- origin used for biofuel (Hiroshi et al., 2017). Moreover, paramylon can be processed into plastics produced with succinic and lactic acids, the ideal bio-ingredients from *E. gracilis* cells under anerobic conditions. This is an environmentally friendly application of *E. gracilis* (Tomita et al., 2016).

Presently, the genetic development of *E. gracilis* for metabolic engineering is limited. Researchers developed traditional strain improvement by transferring DNA vectors for overexpression of genes that encoded the desired proteins. Its performance did not seem ideal based on the historical development of the subject, which could be attributed to both transformation efficiency and lack of complete genome information of *E. gracilis* (Doron et al., 2016). In 2019, Ebenezer et al. (2019) had published the draft nuclear genome of *E. gracilis* with an estimated haploid genome size of about 500 Mb, and a total of 1,266,288 contigs were assembled with a total length of 1.4 Gb, including 1,459 contigs with a length longer than 10 Kb. The longest contig was 16 Kb, and the N50 was only 955 bp. Compared with the previous estimation genome size of *E. gracilis* (1–4 Gb), the new version of the genome size was smaller, and the data can only be used for fundamental analysis. Even the prediction of complete ORF (open reading frame) information about *E. gracilis* was not easy to be provided (Ebenezer et al., 2017). The short reads make it tough to build a complete genome. Other problems such as gene duplication, repetitive sequences, and high complexity of its genome lead to failure in obtaining the full potential of *E. gracilis* in synthesis biology.

To date, microalgae have a remarkable ability to synthesize many value-added natural products that are becoming more significant for academics or industries globally. *E. gracilis,* as a model species, is one of the potential selected microalgae among other industrial *Chlamydomonas reinhardtii*, *Chlorella* sp., and *Haematococcus pluvialis* species , under the increasingly fierce competition with the development of synthetic biology. However, with the genetic studies that focused on analyzing metabolic pathways for the efficient synthesis of high-value products of interest, it is imperative to develop proven bioengineering tools for *E. gracilis* as an industrial biotechnology platform. Thus, we reviewed the progress of the genetic transformation of *E. gracilis*, such as CRISPR and RNA interference, and new ideas for delivery methods of exogenous materials, such as single-cell microinjection and electroporation for algae.

## Harnessing Bioengineering Tools for the Development of *E. gracilis*

### The Progress of the Genetic Transformation of *E. gracilis*

Studies related to the transformation of the nuclear genome of *E. gracilis* were insufficient. The earlier reports of the genetic transformation of *E. gracilis* were focused on the chloroplast genome. Biolistic bombardments coupled with selection biomarkers (i.e., streptomycin, spectinomycin, and neomycin phosphotransferase II) have been used for plastid transformation (Doetsch et al., 2001; Ogawa et al., 2015b). Krajcovic et al. (2011) first reported the successful nuclear genome transformation of *E. gracilis* with a zeocin resistance transformation cassette by electroporation. The *Ble* gene was cloned into the gene of LHCP (light-harvesting chlorophyll a/b binding protein of photosystem) II between the 5′- and 3′- terminal of it so that it could be driven and expressed by the LHCPII promoter. The mutants were detected with *Ble* gene-positive by PCR screening and can be stable for more than a year. Khatiwada et al. (2019) explored the genetic engineering tools to obtain stable *E. gracilis* nuclear transformants *via* constructing plasmid vectors with the selection marker of hygromycin by *Agrobacterium*-mediated transformation, biolistic bombardment, and electroporation techniques. The results showed that only *Agrobacterium*-mediated transformation could produce stable nuclear transformants while the others lost their property after repeated rounds of cultivation. Recently, another *Agrobacterium*-mediated nuclear transformation of *E. gracilis* has been successfully conducted with the selection markers of hygromycin and zeocin. The transformation efficiency was up to 8.26 ± 4.9% after the optimization of co-cultivation parameters (Becker et al., 2021). Although successful nuclear transformation reports were in a small number, those cases showed bright prospects in bioengineering. They provided the genetic tools to produce stable nuclear transformation for *E. gracilis*.

### Antibiotic Resistance and Selection Markers of *E. gracilis*

Antibiotic resistance is critical for transformant screening and selection. However, there is limited study on the sensitivity of *Euglena* to antibiotics. In 2019, Khatiwada et al. introduced vectors with the hygromycin resistance gene *hpt*Ⅱ (encoding hygromycin phosphotransferase Ⅱ) into *E. gracilis* by electroporation, *Agrobacterium*-mediated transformation, and biolistic bombardment. In 2021, Becker et al. explored the sensitivity of *E. gracilis* to a range of antibiotics in liquids and agar plates with different concentrations. The results showed that these antibiotics caused a 55–70% reduction in the growth rate at a concentration of 10 μg/ml of hygromycin and 15 μg/ml of zeocin in liquid culture. The growth was completely inhibited when the concentration was more than 30 μg/ml of hygromycin and zeocin. Under the tested concentrations, the antibiotic kanamycin did not affect *E. gracilis* growth (0, 20, 50, 100, or 200 μg/ml). They also assessed the susceptibility of *E. gracilis* on agar plates with varying concentrations of antibiotics. The lethal concentration of the antibiotics hygromycin and zeocin was 30 μg/ml. No colonies were observed on agar plates more than 30 μg/ml. Those selected markers mentioned previously could facilitate the screening and selection of *E. gracilis*.

### The Delivery Methods of Exogenous Materials for *E. gracilis*

Several exogenous materials, such as DNA, RNA, and RNPs (ribonucleoproteins), are the standard delivery objects for bioengineering. An excellent review on the delivery mode of *E. gracilis*, including protoplast transformation, electroporation, biolistic bombardment, and *Agrobacterium*-mediated transformation, has been summarized elsewhere and will not be dwelt on here (Khatiwada et al., 2020). It is proposed that single-cell microinjection and electroporation could be the efficient molecule delivery ways for *E. gracilis*.

Microinjection is a physical method that uses a glass capillary needle to deliver a small volume of substances such as plasmids and proteins into cells. The materials can be accurately delivered to specific locations of cells, such as the cytoplasm and nucleus. Under the microscope, the manipulated cells can be observed all through the injection procedure, which is helpful for the real-time tracking of injected materials. The microinjection method has been successfully used in mice, zebrafish, rabbits, and much larger livestock such as cattle, sheep, pigs, and other animals as a result of its high efficiency and low cell death rates (Gordon et al., 1980; Hammer et al., 1985; Yuan and Sun, 2009). However, it is rarely conducted in microalgae cells. In 1986, Neuhaus et al. (1986) delivered SV40 DNA and pSV2neo into the nuclei of *Acetabularia* sp. by using the microinjection technique. This single-cell alga could grow vertically to a length of 5 cm. The injected nuclei were implanted into anucleate cell fragments of the same species. The treated cells not only survived but also formed progeny. In 1994, Meindl et al. (1994) had injected phalloidin into the cells of the green alga *Micrasterias denticulata* (the cell size is larger than 100 μm) and observed two types of actin filament systems in the cells, indicating that actin plays a key role in the cell morphogenetic process. However, it is difficult to perform microalgae injection when the size of the cells is less than 20 μm, such as the cells of *E. gracilis* in sphere form. In 1978, Nichols and Rikmenspoel (1978) injected EDTA, EGTA, Zn^2+^, and Mn^2+^ into *E. gracilis* and *C. reinhardtii*, respectively, and explored the relationship between microalgae flagellar movement and divalent cations. Flagellar motility stopped when *E. gracilis* and *C. reinhardtii* were injected with 7 × 10^−14^ and 2 × 10^−14^ L 0.02 M EDTA, respectively. Since then, no delivery of active molecules was reported by microinjection into *Euglena* and the other microalgae with the cell size less than 100 μm.

Single-cell electroporation has been developed based on the single-cell analysis technique, allowing the delivery of ions or molecules into the cell. As we know, millions of cells in a bath mode are analyzed with a low efficiency rate by conventional electroporation. Recently, developed nanofabricated electrodes were able to achieve single-cell electroporation and deliver different types of molecules with high transfection efficiency and high cell survival rate compared to the conventional one (Santra and Tseng, 2016). A modified single-cell electroporation method has been developed for molecular delivery into *E. gracilis* cells (Ohmachi et al., 2016). A variety of molecules, including GFP, Alexa Fluor 488, and exciton-controlled hybridization-sensitive fluorescent oligonucleotide (ECHO) RNA probes have been successfully introduced into living *E. gracilis* cells. This new method realized high transfection efficiency and viability rate after electroporation. Thus, single-cell electroporation techniques of *E. gracilis* will open up a new window for bioengineering manipulation at the single-cell level.

### Gene Editing of *E. gracilis* Meditated by CRISPR-Cas9

In 2013, Zhang’s and Church’s team used the type II system (CRISPR/Cas9) derived from *Streptococcus* to successfully perform genome editing in mammalian cells (Cong et al., 2013; Mali et al., 2013). Since then, CRISPR/Cas9-based genome editing technology has been extensively researched and applied, and many Cas9-based application tools have been developed.

CRISPR/Cas9 is a system based on the endonuclease activity of Cas9 protein and the specific localization function of gRNA for the precise manipulation of foreign genomes. It can be used in many genetic engineering organisms, including animal cells, plant cells, and microalgae cells. The researchers introduced RNPs into *C. reinhardtii* cells by electroporation and successfully obtained CRISPR-/Cas9-induced mutations at the targeted sites of *MAA7*, *CpSRP43*, and *ChlM* genes (Shin et al., 2016). Compared with the first reported mutations induced by transgenic Cas9, the efficiency of delivered Cas9 RNPs has increased by 100 times, significantly improving gene knockout efficiency (Jiang et al., 2014). Researchers have successfully implemented CRISPR/Cas9 gene-editing technology in microalgae such as *Nannochloropsis spp*, *Phaeodactylum tricornutum*, and *Thalassiosira pseudonana* in the past 10 years (Hopes et al., 2016; Nymark et al., 2016; Wang et al., 2016)*.* Recently, Nomura et al. (2019), Nomura et al. (2020) had successfully introduced Cas9 RNPs into *E. gracilis* through electroporation. Through a non-homologous end-repair mechanism, the mutation efficiency of the targeted gene *EgGSL2* was as high as 90.1%. These two are the only research articles on the successful gene editing of *E. gracilis* using the CRISPR/Cas9 system, and no successful reports have followed yet.

### Application of RNA Interference Technology in *Euglena* Cells

RNA interference (RNAi) is a phenomenon of the specific degradation of homologous mRNA induced by double-stranded RNA (dsRNA). It is a conservative self-defense mechanism to resist transgenes and foreign viruses in the evolutionary process. The homologous dsRNA of the target gene is artificially constructed and introduced into the cell to silence the expression of the target gene. In 2002, RNAi technology was used to study the function of a nuclear gene (blue light receptor-photosensitized adenylate cyclase, PAC) in *E. gracilis*, resulting in significant reduction of PAC content in the cells (Iseki et al., 2002). Subsequently, RNAi technology has been frequently used in the gene function verification of *E. gracilis*. For instance, RNAi was conducted to silence calmodulin-related genes to explore the influence of calmodulin-related genes on the gravity axis of *E. gracilis* and its movement mode (Häder et al., 2005; Daiker et al., 2010; Nasir et al., 2018). Nasir focused on the flagellin EgPCDUF4201 and its interaction with calmodulin CaM.2 (Nasir et al., 2018). In subsequent research, the focus shifted to the basic metabolic processes of *E. gracilis*, especially the metabolites such as wax and paramylons. Since 2014, more than ten researchers have used RNAi technology to explore a variety of basic metabolic processes, including photosynthetic pigments, redox processes, wax metabolism pathways, and paramylon metabolism processes, and search for the connection of genes and metabolic processes. So far, many studies about gene function have been carried out in *E. gracilis* by RNAi technology (Table 1). These genes are involved in *E. gracilis*’ primary metabolism, light response, and metabolite synthesis. The methods of introducing DNA and gene cloning used in these documents can be optimized to study the genetic transformation of *E. gracilis*.

**TABLE 1:** Studies on the gene function of *E. gracilis* by RNAi technique.

Gene	Full name	Main function	Reference
*ALase*	Aldonolactonase	Catalyzes the synthesis of ascorbic acid	Ishikawa et al. (2008)
*APXs*	Ascorbate peroxidase	Participates in the metabolism of reactive oxygen species	Ishikawa et al. (2010)
*Cals*, *CaMs*	Calmodulins	Transient receptor potential channel, related to the gravity axis	Häder et al. (2005), Häder et al. (2009), Daiker et al. (2010), Nasir et al. (2018)
*CRT B*	Phytoene synthase	Key enzymes for synthesizing phytoene	(Kato et al., 2017; Tamaki et al., 2020)
*CYP97H1*	Carotene hydroxylase	Involved in the hydroxylation of *β*-carotene	Tamaki et al. (2019)
*FBPase*	Fructose-1,6-bisphosphatase	Key enzymes of Calvin cycle	Ogawa et al. (2015a), Ogawa et al. (2015b)
*GSL2*	Glucan synthase–like 2	Key enzymes in the synthesis of paramylon	Tanaka et al. (2017)
*KAT*	3-ketoacyl-CoA Thiolase	*Euglena* mitochondrial-fatty acid-condensing enzyme	Nakazawa et al. (2015)
*NTRs*	NADPH-dependent thioredoxin reductase, NADPH	Redox reaction regulator	Tamaki et al. (2015), Ozasa et al. (2017)
*Prxs*	Peroxiredoxins	Participate in the metabolism of reactive oxygen species	Tamaki et al. (2014), Ozasa et al. (2017)
*PAC*	Photoactivated adenylyl cyclase	*Euglena* blue light receptor, related to phototaxis	Iseki et al. (2002), Ntefidou et al. (2003), Ozasa et al. (2017)
*PKA*	Protein kinase A	Participates in signal transduction of *Euglena* phototaxis and gravity axis	Daiker et al. (2011)
*PNO*	Pyruvate: NADP^+^ oxidoreductase	Participates in the fermentation of *Euglena* wax fat	Nakazawa et al. (2017)
*2-ogdc*	2-oxoglutarate decarboxylase	Key enzymes of the tricarboxylic acid cycle	Nakazawa et al. (2017)
*RGA lU*	UDP-glucose pyrophosphorylase	Involved in the synthesis of paramylon	Muchut et al. (2021)
*STDs*	Starch degradation	*Euglena* growth and proliferation- related protein	Kimura and Ishikawa, (2018)
*TERs*	Trans-2-enoyl-CoA reductases	Play the role of transferring hydrogen in the metabolic process	Tomiyama et al. (2019)
*WSDs*	Wax ester synthase	Key enzymes of *Euglena* wax fermentation	Tomiyama et al. (2017)

## Discussion

**FIGURE 1:**
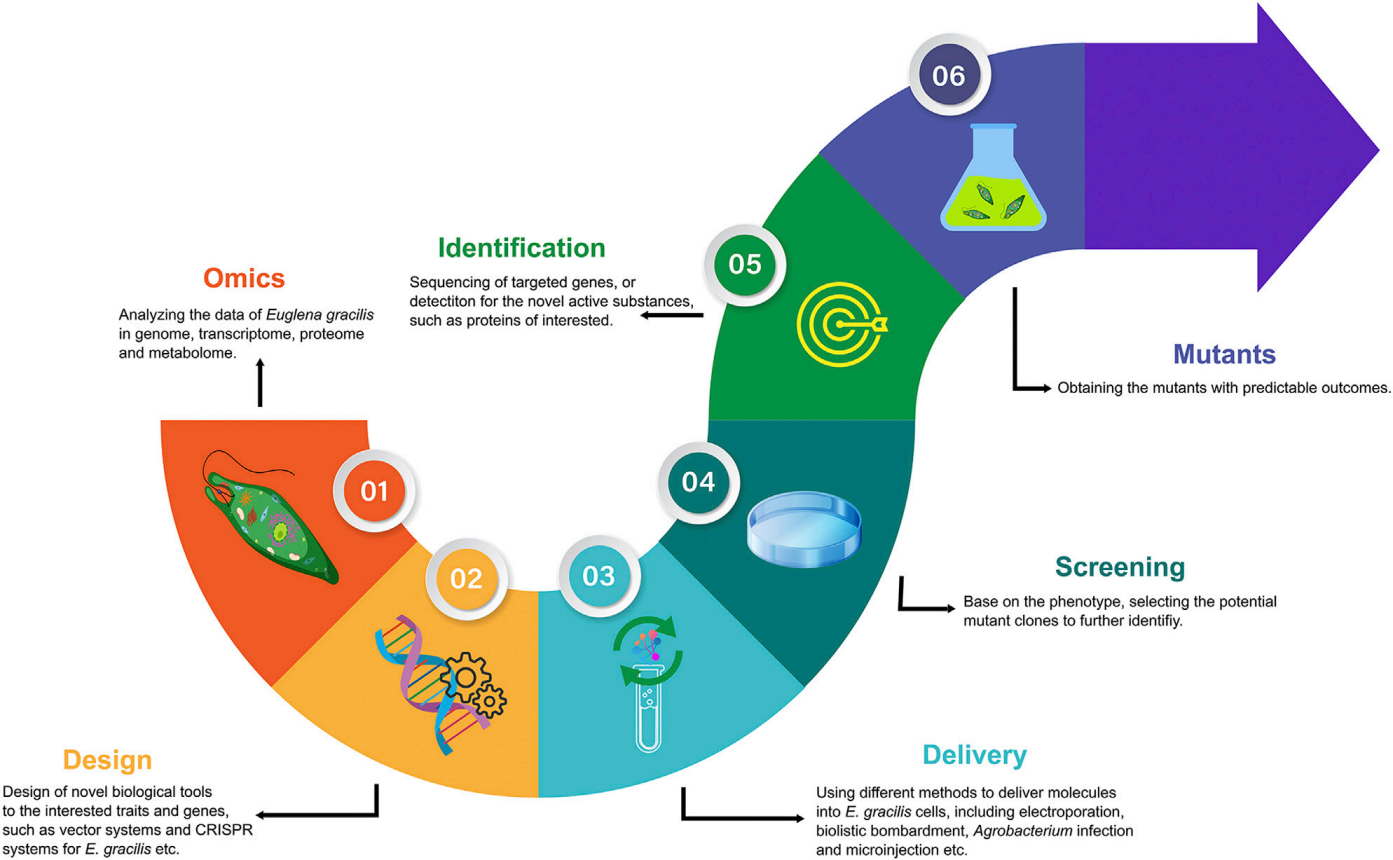
Schematic flowchart of a bioengineering workflow on synthetic biology for *E. gracilis*.

Presently, synthetic biology on microalgae has followed the workflow of analyzing the omics data, designing the transformation systems, delivering exogenous molecules, and screening, identification, and obtaining mutants with predictable outcome (Figure 1). The excavation of genetic information to assist metabolic engineering of *E. gracilis* is essential to improve the subsequent design of novel biological tools. The design of promoters, targeted sites of interested genes, transcription terminators and regulators, plasmids, etc., is mainly based on genome integrity and quality when performing bioengineering works. However, the number of nuclear transformation reports for *E. gracilis* is minimal because of lack of complete genome information. To some extent, the draft genome of *E. gracilis* has adequately promoted the promoters or other elements to develop. For the gene function identifying of *E. gracilis*, the tools of RNAi that can silence the gene function were recommended. Because the number of the relevant cases was much greater than those of the nuclear transformation, it means a high efficiency rate but will not cause permanent phenotype (Table 1). The CRISPR technique offers the opportunity to obtain mutants with predictable outcomes permanently. However, there are only two research articles on *E. gracilis* regarding CRISPR/Cas9 so far [Nomura et al. (2019), Nomura et al. (2020)]. The efficiency rate was as high as approximately 70–90% using electroporation without a knock-in selected marker gene, which was hardly reproducible by other teams, including ours, since 2020. The reasons may be difficulty in optimizing the parameters by electroporation or lack of the knock-in selected marker for mutant screening. For example, Shamoto et al. (2018) have obtained a 55% gene editing efficiency rate of *fap70* in *C. reinhardtii* by using a knock-in paromomycin-resistance gene as a selected marker, which was much higher than that in the report of CRISPR/Cas9 in *C. reinhardtii* without selected markers by delivering RNPs using electroporation (Baek et al., 2016). *Agrobacterium*-mediated transformation may be the most efficient way among electroporation and biolistic bombardment for obtaining stable mutants in bulk cells of *E. gracilis* based on Khatiwada et al. (2019), and also can be supported by Becker et al. (2021). Nevertheless, with the rise of single-cell analysis technologies, microinjection and single-cell electroporation will be powerful delivery tools to introduce exogenous molecules into microalgae cells with high transformation efficiency and high cell viability. We conducted the CRISPR/Cas9 genome editing on *E. gracilis* and successfully knocked out the *Crtp1* gene by microinjection with a high efficiency rate (16.7%) (Chen et al., 2021). However, the following problems still limit its wide use: expensive equipment, experienced operators, and time consumption. For screening, reducing the workload of screening was determined by designed steps at the beginning. A significant phenotype would be helpful in picking up the transformants conveniently and quickly. Finally, the sequencing of targeted genes can be used to identify mutants further, and other methods such as Western blot or HPLC (high-performance liquid chromatography) are the ideal means to analyze the quality and quantity for the designed active substances, such as the proteins, lipids, and pigments.

In summary, *E. gracilis,* with its special biological characters, has excellent potential for academic and economic values. However, stable nuclear transformation is still a problem today. We are happy to see the significant contributions of researchers from all over the world when the “1st Annual International Congress on Euglenoids” has been successfully held and closed in London in 2021, and we believe that the genetic conversion of *Euglena* has the following research directions: 1) to complete the genome sequencing of *Euglena*; 2) to elucidate the replication mechanism of DNA, internal and external DNA; 3) to find out high-expression promoters, codon optimization, and intron embedding; 4) to construct more efficient *Euglena* selection biomarkers; and 5) to develop CRISPR-based genome editing systems for RNAi and other genetic engineering tools that *Euglena* has not reported.

Recently, gene engineering targeted genes mainly focusing on commercial bioproducts, with single gene/enzyme manipulations: 1) synthesis or degradation of bioactive compounds and high valued-added ingredients such as paramylon, wax, fatty acids, and carotenoids; 2) reactive oxygen species scavengers; and 3) receptors related to phototaxis and gravity axis. In the near future, *E. gracilis* might become a model synthetic microbial chassis for exploring photosynthesis; retrograde pathways as the essential communication between the nucleus and the two DNA-containing organelles, synthetic chloroplast; flagellar elongation and shortening; and epigenetic components and mechanisms, including DNA methylation, histone modifications, and microRNAs. Thus, with the improvement of bioengineering tools, the synthetic biology research of *Euglena* will achieve enormous progress, and genetic engineering transformation will significantly promote the industrial application and scientific progress of these unique protists.
